# The Resurgence of Syphilis: A 20-Year Evaluation of Epidemiological Trends and Serological Test Performance Using Rapid Plasma Reagin and Indirect Hemagglutination Assays

**DOI:** 10.3390/medicina61081491

**Published:** 2025-08-20

**Authors:** Melda Payaslıoğlu, İmran Sağlık, Esra Kazak

**Affiliations:** 1 Department of Medical Microbiology, Faculty of Medicine, Bursa Uludağ University, 16000 Bursa, Turkey; imransaglik@uludag.edu.tr; 2 Department of Infectious Diseases and Clinical Microbiology, Faculty of Medicine, Bursa Uludağ University, 16000 Bursa, Turkey; eskazak@uludag.edu.tr

**Keywords:** syphilis, Rapid Plasma Reagin (RPR), Indirect Hemagglutination Assay (IHA), epidemiology, disease resurgence

## Abstract

*Background and Objectives*: This retrospective single-center study aimed to evaluate the epidemiological, clinical, and laboratory characteristics of syphilis cases diagnosed at our hospital between 2005 and 2024, with a focus on the performance of serological tests used for diagnosis. The study also sought to characterize changing epidemiological trends of syphilis over this 20-year period. *Materials and Methods*: Data from 671 patients with confirmed syphilis diagnoses were retrospectively analyzed. Demographic information, transmission routes, co-infection status, and serological test results—including Rapid Plasma Reagin (RPR) and Indirect Hemagglutination Assay (IHA)—were evaluated. Statistical analyses were performed using chi-square and Fisher-based tests, with Bonferroni correction applied for multiple comparisons *Results*: Of the 671 cases, 74.6% were male and 25.4% female, with the highest incidence in the 22–41 age group. The number of diagnosed cases increased approximately 6-fold after 2016 compared to the preceding years. Unprotected sexual contact was the most common transmission route. HIV co-infection was present in 32.6% of cases, predominantly in males. Significant differences in RPR and IHA titers were observed across clinical stages of syphilis, with notably higher titers in late latent and neurosyphilis cases. *Conclusions*: The 6-fold increase in syphilis diagnoses since 2016, alongside a high rate of HIV co-infection, underscores the need for targeted prevention and screening programs for high-risk populations. Serological testing remains essential for diagnosis and disease monitoring.

## 1. Introduction

Syphilis, caused by *Treponema pallidum* subsp. *pallidum*, remains a persistent and re-emerging sexually transmitted infection (STI) worldwide despite being curable with appropriate antibiotic treatment [1]. According to the 2024 World Health Organization (WHO) report, syphilis continues to impose a significant burden in certain regions, with increasing incidence particularly among key populations such as men who have sex with men (MSM), individuals with HIV, and pregnant women [2].

If left untreated, syphilis can progress through distinct clinical stages—primary, secondary, latent, and tertiary—each with characteristic features. The latent stage, which is asymptomatic, is further classified into early latent (within the first year of infection), late latent (after one year), and latent of unknown duration. Tertiary manifestations, although less common today, may lead to severe neurological or cardiovascular complications [3].

In clinical practice, the diagnosis and follow-up of syphilis heavily rely on serological testing. The current diagnostic approach integrates non-treponemal tests such as Rapid Plasma Reagin (RPR), which reflect disease activity and treatment response, with treponemal tests such as the Indirect Hemagglutination Assay (IHA), which detect antibodies to *T. pallidum* regardless of treatment status [4]. Despite technological advancements, challenges persist in interpreting serological titers, particularly in cases of reinfection, coinfection (e.g., HIV), and serofast states where titers do not decline as expected after therapy.

We hypothesize that analyzing the performance and interpretability of RPR and IHA titers over a 20-year period can provide meaningful insight into the diagnostic utility and epidemiological trends of syphilis in a tertiary care setting. Such long-term evaluations are essential in 2025 and beyond, especially in regions where test algorithms and patient profiles have evolved but commercial test variability persists.

This retrospective study aims to assess the demographic, clinical, and serological characteristics of syphilis cases diagnosed at a single tertiary hospital in Türkiye between 2005 and 2024. It further seeks to describe long-term epidemiological trends and evaluate the stage-specific utility of RPR and IHA testing within this cohort.

## 2. Materials and Methods

This retrospective descriptive study was conducted using data from patients diagnosed with syphilis who presented to the inpatient and outpatient clinics of our hospital between 2005 and 2024.

### 2.1. Patient Selection

This retrospective study was conducted at Bursa Uludağ University Faculty of Medicine, a tertiary care university hospital serving Bursa and its surrounding regions. The majority of patients diagnosed with syphilis between 2005 and 2024 were seen in the Infectious Diseases inpatient ward and outpatient clinic. However, patients were also referred from other departments, including Dermatology, Obstetrics and Gynecology, Neurology, Ophthalmology, and others, reflecting the multisystem nature of the disease. During this period, the regional population increased, potentially leading to a higher number of patient admissions. All syphilis cases definitively diagnosed by infectious disease specialists and confirmed by laboratory testing (*n* = 671) were included in the study. No cases were excluded due to missing data or incomplete records. Data on mode of transmission and syphilis stage were available for all 671 patients; no missing values were present for these variables. Additionally, the hospital’s bed capacity changed over time; notably, during the COVID-19 pandemic, a significant portion of beds in the Infectious Diseases ward was allocated to COVID-19 patients, temporarily reducing inpatient admissions for other conditions, including syphilis. Diagnosis was based on clinical evaluation and positive serologic test results, including IHA and/or Venereal Disease Research Laboratory (VDRL)/RPR tests. Data from 671 patients were retrospectively analyzed using the hospital’s electronic medical record system. As the study focused solely on patients with a confirmed syphilis diagnosis, the impact of fluctuations in hospital bed availability and regional patient population on the final sample is considered minimal.

Demographic characteristics such as age, gender, marital status, region of residence, and mode of transmission were examined, along with the presence of HBV, HCV, and HIV coinfections. Routine testing for HIV, HBV, and HCV was performed in all patients diagnosed with syphilis in accordance with the institutional infectious disease protocol. HBV coinfection was defined based on HBsAg positivity; individuals who were HBsAg negative but anti-HBc positive were not considered coinfected. HCV coinfection was defined as anti-HCV positivity. In addition, the clinical stages of syphilis and corresponding serologic test results were evaluated. Syphilis stages were classified as primary, secondary, latent, and neurosyphilis, in accordance with the CDC’s Sexually Transmitted Diseases Treatment Guidelines [5]. In accordance with these guidelines (2021), latent syphilis cases were further subcategorized as early or late latent. Early latent syphilis was defined as asymptomatic infection acquired within the previous 12 months, supported by one or more of the following: (i) a documented negative serological test result within the past year; (ii) clinical history of symptoms consistent with syphilis within the prior year; (iii) known exposure to a syphilis-positive partner in the past 12 months; or (iv) epidemiological linkage to a recent outbreak. Cases without reliable documentation or clinical evidence to determine the duration of infection were classified as late latent syphilis by default, in accordance with guideline recommendations. Serological test results used in this study were based on the earliest available tests performed prior to treatment initiation, ensuring the most accurate representation of baseline antibody titers.

This study analyzed confirmed syphilis cases with complete serological data and clinical diagnoses established by the Infectious Diseases Department. Laboratory evaluation focused on diagnostic and epidemiological parameters. Clinical staging was determined based on evaluations by specialist physicians and multidisciplinary case discussions. Although a formal inter-rater reliability analysis was not performed, consistency in staging was ensured through these case reviews.

### 2.2. Laboratory Tests

While the basic principles of the serological tests (IHA and/or RPR/VDRL) employed throughout the study period remained consistent, occasional changes occurred in the commercial kit brands used. Due to the retrospective nature and the long duration of the study, detailed information on all commercial kit brands used throughout the 20-year period could not be fully retrieved. However, all serological tests were performed using CE-marked commercial kits approved for clinical use. According to internal laboratory documentation, no significant analytical performance discrepancies were reported between different kit brands used over the study period. Nevertheless, all tests were conducted in accordance with standard protocols and evaluated under the laboratory’s internal quality control procedures. Serologic test results were classified based on antibody titer levels. For the Rapid Plasma Reagin (RPR) test, titers of 1:1–1:2 were considered low, 1:4–1:16 moderate, and ≥1:32 high. For the Syphilis Indirect Hemagglutination Assay (IHA), based on the laboratory’s reference ranges, titers of 1:10–1:40 were considered low, 1:80–1:160 moderate, and ≥1:320 high. The cutoff values used for the IHA test were based on the manufacturer’s instructions and included in the test kit’s procedural guidelines. Borderline titers were interpreted according to these predefined reference ranges. These classifications were used to support diagnostic evaluation and disease activity assessment.

We would like to clarify that in our study, VDRL testing was performed exclusively on cerebrospinal fluid (CSF) samples, comprising only 13 neurosyphilis cases. For the serological analysis correlating antibody titers with syphilis stages, only RPR results from blood samples were included. Therefore, VDRL and RPR titers were not grouped together, and their quantitative differences were taken into account during the analysis.

Although the study spans a 20-year period, there were no major changes in the diagnostic testing algorithm implemented at our institution. The only variation involved the brands of commercially available test kits. Throughout the entire period, the standard testing algorithm consistently followed the traditional sequence: RPR as the initial screening test, followed by IHA for confirmation. A reverse testing algorithm (starting with treponemal tests) is used exclusively in our affiliated blood center for blood donor screening purposes; however, these data were not included in this study, as the blood center’s database is separate from the hospital’s clinical patient records.

### 2.3. Statistical Analysis

Categorical variables were compared using Pearson chi-square, Fisher’s exact, and Fisher–Freeman–Halton tests between groups. Cramer’s V value was given as an effect size measure for categorical analysis. The Bonferroni test was used as a multiple comparison test. Wilcoxon rank-sum test was used for comparison of the antibody titers measured by IHA and RPR tests. Statistically, the significance level was accepted as alfa = 0.05. Statistical analyses were performed with IBM SPSS 29.0.2.0 (IBM corp. Released 2023. IBM SPSS Statistics for Windows, Version 29.0.2.0, Armonk, NY, USA: IBM Corp.).

### 2.4. Ethical Approval

Approval for the study was obtained from the Bursa Uludağ University Faculty of Medicine Clinical Research Ethics Committee (Decision No: 2025/12-29, Date: 25 June 2025).

## 3. Results

**Figure 1:**
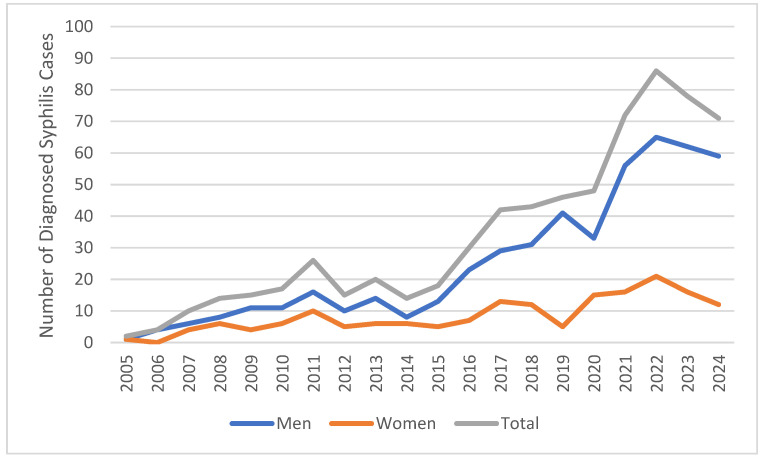
Changes in the number of syphilis cases by year and gender (2005–2024).

Data from 671 patients diagnosed with syphilis who presented to our hospital between 2005 and 2024 were analyzed. Of these, 501 (74.6%) were male and 170 (25.4%) were female. An overall increase in syphilis cases was observed over the years, with a particularly notable rise since 2016. The proportion of male patients remained higher than that of female patients throughout the study period (Figure 1).

**Figure 2:**
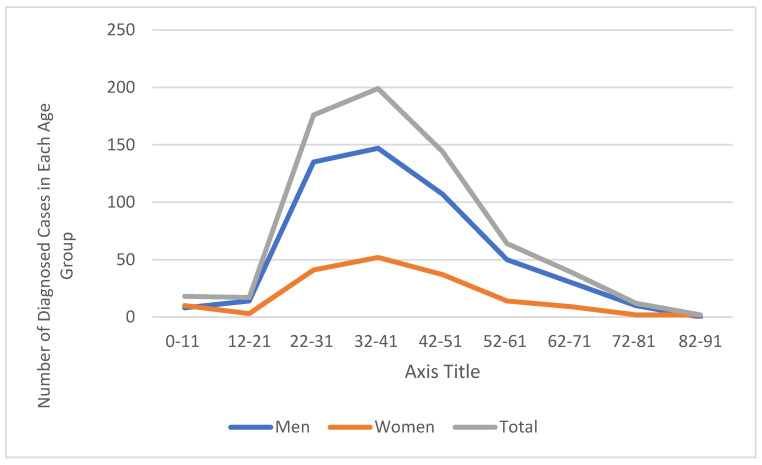
Distribution of syphilis cases by age group and gender (*n* = 671).

When examining the distribution of cases by age group, the highest number was observed in the 22–41 age range (Figure 2). Among the 176 cases aged 22–31, 135 were male (76.7%) and 41 were female (23.3%), while in the 32–41 age group, 147 of 199 cases were male (73.9%) and 52 were female (26.1%). These two age groups together accounted for approximately 56.0% of all cases. A statistically significant upward trend was found in the proportion of male cases over the years (Cramer’s V effect size = 0.252; *p* = 0.002), with this trend being particularly prominent in the 2019 data.

Overall, syphilis cases aged between 22 and 51 (*n* = 519) constituted 77.3% of the total cases. In the 42–51 age group, 144 cases were identified, of whom 74.3% were male. The number of cases was lower in other age groups. Notably, 18 cases (8 males, 10 females) were identified in the 0–11 age group, which were considered for evaluation of congenital syphilis. In older age groups (62 years and above), the number of cases gradually decreased. No significant differences were found in the distributions by age group and gender.

Epidemiological data of the cases are presented in Table 1. When transmission routes were analyzed, it was observed that the majority of cases were transmitted through unprotected sexual contact (UCS). UCS was identified as the transmission route in 476 cases (70.9%), with a significantly higher number of males (*n* = 376) than females (*n* = 100) in this group (Cramer’s V effect size = 0.204; *p* < 0.001). The second most common category was unknown transmission route, accounting for 144 cases (21.5%). Other transmission routes, such as injection/substance use (*n* = 15, 2.2%), blood transfusion (*n* = 18, 2.7%), and mother-to-child transmission (congenital transmission) (*n* = 18, 2.7%), were reported less frequently.

Regarding residence, the majority of patients lived in Bursa (*n* = 610, 90.9%). Among the 61 patients (9.1%) from outside Bursa, most were women (44 women, 17 men). No significant difference was found by gender in terms of residence (Cramer’s V effect size = 0.018; χ^2^ = 0.228; df = 1; *p* = 0.633). Marital status analysis revealed that over half of the patients were single (*n* = 337, 50.2%). Most male patients were single (*n* = 273, 54.5%), whereas the majority of female patients were married (*n* = 106, 62.4%) (Cramer’s V effect size = 0.204; *p* < 0.001).

**Figure 3:**
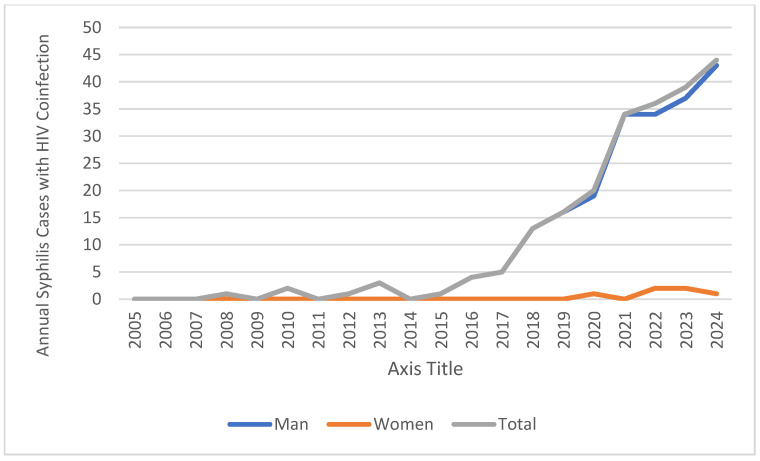
Syphilis–HIV coinfections by year.

Serological data for other sexually or parenterally transmitted infections, such as HIV, HBV, and HCV, were examined in 671 syphilis cases included in the study. HIV was the most common coinfection, detected in 219 patients (32.6%). The majority of these cases were male (*n* = 213, 97.3%) (Cramer’s V effect size = 0.362; *p* < 0.001), with only six female patients (2.7%) showing coexistence of HIV and syphilis. This suggests that syphilis–HIV coinfection is particularly common among males, possibly among men who have sex with men (MSM). HCV positivity was detected in 17 cases (2.5%), and HBV positivity in 8 patients (1.2%) (Figure 3, Table 2).

Between 2005 and 2024, HIV coinfection steadily increased among syphilis cases admitted to our hospital (Figure 3). In the early years (2005–2015), coinfection cases were rare or often nonexistent. However, a significant upward trend has been observed since 2016, particularly among male patients. HIV coinfection was observed at a much lower rate in female patients (six cases in total).

**Figure 4:**
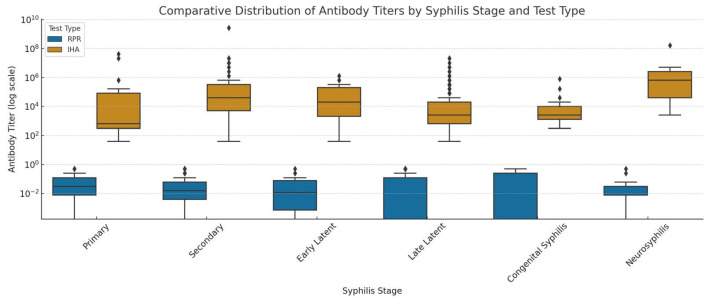
Comparative distribution of antibody titers measured by IHA and RPR tests across clinical stages of syphilis shown as box-and-whisker plots. The plots highlight differences in titers related to disease progression and test-specific characteristics. In the box plots, the horizontal line inside the box represents the median value; the box itself indicates the interquartile range (IQR), spanning from the first quartile (Q1) to the third quartile (Q3). The whiskers extend to the minimum and maximum values within 1.5 times the IQR, excluding outliers. Points outside these whiskers represent outliers. Colors denote test types: orange for IHA and blue for RPR. The figure is intended solely for visual presentation and does not include any statistical tests or *p*-values.

In our study, antibody titers measured by both IHA and RPR tests significantly correlated with the clinical stages of syphilis (*p* < 0.001) (Figure 4). Low IHA titers were observed in primary syphilis cases (*n* = 29), while moderate to high titers were more frequent in secondary cases (*n* = 110). In early latent cases (*n* = 16), IHA titers remained mostly low to moderate. In late latent cases (*n* = 485), titers were broadly distributed and notably elevated, particularly at 1/320, 1/640, 1/1280, and 1/2560 dilutions. Very high titers (>1/20,480) were seen in neurosyphilis cases (*n* = 13). In congenital syphilis (*n* = 18), IHA titers ≥ 1/320 were detected.

Similarly, RPR titers showed stage-dependent variation. Among 671 cases, primary syphilis patients (*n* = 29) had RPR titers spread across a wide range with no negative results. In secondary cases, high titers (≥1/128) were more common. Early latent cases showed mostly low titers. In late latent cases, both negative and low positive results at varying dilutions were observed. In neurosyphilis and congenital syphilis, RPR positivity was less frequent and scattered across a wide titer range.

**Figure 5:**
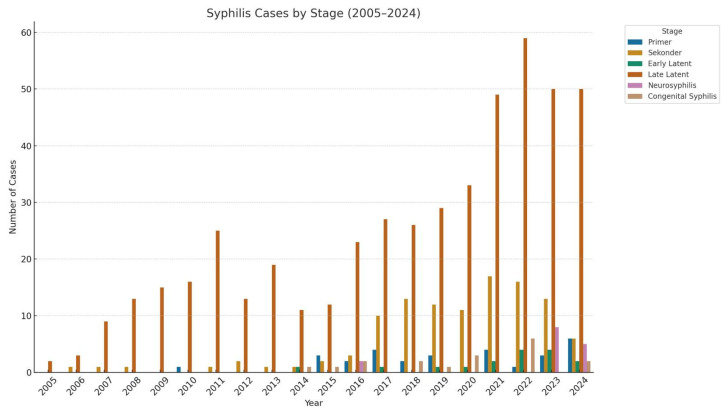
Annual distribution of syphilis cases by clinical stage in a 20-year period (2005–2024).

Figure 5 shows a notable increase in late latent syphilis cases starting from 2011, reaching a peak of 50 cases in 2024. This may indicate delayed diagnoses and missed opportunities for early detection. Secondary syphilis cases also rose significantly after 2015, particularly between 2017 and 2022. Primary syphilis remained relatively low but increased in 2023 and 2024, with a maximum of six cases in the final year. Neurosyphilis and congenital syphilis cases became more prominent in recent years. The rise in neurosyphilis (eight cases in 2023) and the reappearance of congenital syphilis in 2022 and 2024 point to gaps in both adult treatment and prenatal screening programs.

## 4. Discussion

The most significant finding of this study is the marked increase in the number of patients diagnosed with syphilis over the past 20 years, particularly during the last decade. This upward trend indicates a re-emergence of syphilis and highlights its growing importance as a public health concern. Our long-term epidemiological analysis, conducted at a tertiary care university hospital in Türkiye, underscores that syphilis remains a relevant and challenging infectious disease, with increasing incidence suggesting gaps in prevention, diagnosis, or treatment strategies.

Despite the knowledge of the causative agent and its transmission routes, as well as the availability of definitive treatment, the incidence of syphilis continues to rise globally and in our country, remaining a significant public health concern [6,7,8]. Although national surveillance data are available, detailed long-term epidemiological studies from Türkiye are relatively limited. According to syphilis statistics published by the Ministry of Health’s General Directorate of Public Health, the number of reported cases increased from 502 in 2015 to 2801 in 2021 [9]. However, the reported annual incidence rate of 3.35 per 100,000 population is believed to be an underestimation, likely due to underdiagnosis and underreporting.

Recently, several valuable studies have been published from Türkiye, focusing on specific subgroups such as individuals living with HIV [10,11] and neonates born to infected mothers [12]. While these reports provide important insights, long-term trend analyses involving broader populations and serological data remain scarce. Our study aims to contribute to filling this gap by providing a comprehensive 20-year overview of syphilis trends and diagnostic patterns in a tertiary hospital setting.

In our study, a significant increase was observed in syphilis cases admitted to our hospital between 2005 and 2024, with a particularly notable rise starting from 2016. This upward trend is consistent with data from both our country and across Europe [8,9,13]. According to the 2022 report by the European Centre for Disease Prevention and Control (ECDC), a 41% increase in syphilis cases was reported in EU/EEA countries between 2018 and 2022, with a 34% increase occurring between 2021 and 2022 alone [13].

Additionally, changes in case-finding strategies, such as expanded prenatal screening programs and targeted interventions among men who have sex with men (MSM) populations, may have contributed to the observed increase in syphilis diagnoses. These factors should be considered alongside true incidence changes when interpreting epidemiological trends.

During the COVID-19 pandemic, the number of reported syphilis cases decreased in 2020 due to reduced syphilis testing and clinical visits in many countries, but an increase in cases was observed again in 2021. This trend was confirmed in the ECDC’s 2021 report, which noted an 87% increase in cases in 2021 following the decline during the pandemic year 2020 [14]. Similar findings were reported in our study and in a study conducted by Yürüyen C. et al. in our country [15].

In our study, male patients constituted 74.6% of syphilis cases, and this gender distribution remained predominant across all age groups; this is consistent with global data. According to the global GBD 2019 analysis, the incidence of syphilis was significantly higher in males than in females, with the highest incidence among males aged 24–29 and females aged 20–24 [16]. A similar trend is observed in European data; the 2019 ECDC report shows that the proportion of male cases in the EU/EEA region is approximately nine times that of females, with cases most concentrated in the 25–34 age group [17]. More recent 2022 data indicate that this trend continues, with the male case rate eight times higher than females and the highest number of cases again in the 25–34 age group [13]. Similarly, in our data, the male case rate was 74.6%, with cases particularly concentrated in the 22–41 age group (56%), where the proportion of males ranges between 74% and 77%.

In our study, the vast majority of syphilis cases (70.9%) were attributed to transmission through unprotected sexual intercourse. This aligns with World Health Organization data, which report congenital (transplacental) and parenteral transmission as less common routes [18]. In our dataset, injection/substance use (2.2%), blood transfusion (2.7%), and congenital transmission (2.7%) were also identified as transmission routes, albeit at lower rates. Furthermore, our 18 congenital syphilis cases underscore the clinical importance of mother-to-child transmission and highlight the necessity of prenatal screening programs.

HIV and syphilis affect similar patient populations, and coinfection is common. Syphilis is associated with an increased risk of HIV transmission, potentially due to a higher incidence of genital ulcers, and it may present with atypical features in HIV-positive patients [19]. Therefore, the detection and treatment of syphilis can help reduce HIV transmission [19]. HIV/syphilis coinfection rates vary by country, sexual orientation, and educational status [19]. According to the ECDC 2021 Syphilis Epidemiological Report, among 17 countries reporting HIV coinfection status, 15% of cases were HIV-positive (known or newly diagnosed), 54% were HIV-negative, and 31% were unknown [14]. In a study evaluating 1852 early syphilis cases in Washington between 2015 and 2019, HIV coinfection was detected in 43% of cases [20]. Marchese et al. [21] found HIV coinfection in 52% of 663 syphilis cases in Italy. Studies conducted in our country have reported HIV coinfection rates among syphilis patients ranging from 1% to 25% [22,23]. Both domestic and international studies have found that the proportion of male patients with HIV/syphilis coinfection ranges from 92.4% to 100% [19,21,23,24]. In our study, HIV coinfection was detected in 32.6% of syphilis patients; notably, almost all of these cases were male (97.3%). These findings demonstrate that syphilis and HIV infections have been increasing, particularly among male patients, in recent years. This supports the concurrent spread of both infections due to shared risk factors and highlights the importance of screening for HBV, HCV, and syphilis during HIV screening in clinical practice, particularly for planning simultaneous treatment management.

Serological tests such as IHA and RPR, commonly used alongside clinical and historical evaluation, remain essential tools for both the diagnosis and monitoring of syphilis [1]. Our findings confirm that antibody titers measured by these tests significantly correlate with the clinical stages of the disease (Figure 4), offering valuable insights into disease activity and progression.

In primary syphilis cases, low IHA titers and the absence of negative RPR results highlight the high sensitivity of non-treponemal tests even at early stages [1,25,26]. The secondary stage, characterized by high antigen load, showed a strong serologic response, with moderate-to-high IHA titers and RPR titers frequently at or above 1/128, aligning with previous studies and CDC diagnostic guidelines [1,5,25].

In early latent cases, titers from both tests generally remained low to moderate, reflecting the asymptomatic but immunologically active nature of this stage. Conversely, in late latent syphilis, IHA titers were widely distributed and frequently elevated—particularly between 1/320 and 1/2560—suggesting persistent treponemal antigen stimulation. RPR results in this group were more heterogeneous, with both negative and low-positive titers observed, consistent with the known reduction in non-treponemal test sensitivity over time [1,26].

Neurosyphilis cases were notable for their very high IHA titers (>1/20,480), which may reflect sustained immune activation within the central nervous system. However, serologic results in this group must be interpreted in conjunction with clinical signs and cerebrospinal fluid findings, as serology alone is insufficient for definitive diagnosis [1,25,26].

In congenital syphilis, the detection of IHA titers ≥1/320 and low-to-moderate RPR titers underscores the importance of comprehensive prenatal screening and careful postnatal follow-up. Although non-treponemal titers tend to be lower in this group, their presence remains diagnostically relevant, particularly when interpreted in parallel with maternal serology.

Overall, our findings indicate that neither test alone is sufficient to accurately determine the stage of the disease. However, the combined evaluation of IHA and RPR tests can provide valuable support for clinical assessment, especially in settings where molecular diagnostic methods are unavailable or limited.

Although our study did not include treatment outcomes or long-term follow-up, the stage-specific serological profiles we present may serve as an important reference for the development of local diagnostic and monitoring protocols in the future. Stage-specific antibody titer data, such as IHA levels, can also offer valuable guidance in refining local treatment and follow-up algorithms. While serological titers alone are not definitive markers for diagnosing neurosyphilis, higher titers in certain disease stages may indicate increased risk, warranting consideration of earlier diagnostic interventions such as lumbar puncture. This approach aligns with clinical guidelines that emphasize comprehensive evaluation including serology, clinical findings, and risk factors. To optimize patient management, especially in local settings, further prospective studies are needed to validate titer thresholds.

Nevertheless, given the retrospective nature of our study, possible errors in stage classification, unknown prior history of syphilis in patients, and changes in test kits over time, these findings should be interpreted with caution. The stage-specific serological patterns observed in our dataset should be regarded not as definitive conclusions but as preliminary observations to guide more controlled, prospective research. Comprehensive prospective studies are required to determine whether antibody titer thresholds can reliably contribute to staging, treatment decisions, or follow-up strategies.

While our findings contribute significantly to the long-term understanding of syphilis epidemiology in Türkiye, it should be noted that the demographic characteristics of Bursa province are unique, which may limit the generalizability of our findings to the entire country. Therefore, caution is warranted when extrapolating these results to a national level. It is important to interpret them within the broader context of international surveillance challenges.

In countries such as Poland, health insurance data indicate that the actual number of syphilis cases may be nearly twice the number reported by national epidemiological surveillance systems [27]. Similarly, a retrospective model-based analysis in Germany, combining statutory health insurance claims and blood donor seroprevalence data, estimates syphilis burdens that exceed traditional notification system figures [28]. These findings emphasize that underreporting remains a concern even in countries with well-established surveillance infrastructure. This reinforces the likelihood that national incidence rates may underestimate the true prevalence. Long-term institutional datasets like ours are therefore essential for bridging the gap between reported and actual disease burden, and for guiding evidence-based public health strategies.

Serological titers offer important insights into the stage and activity of syphilis; however, the diagnosis—especially of late latent and neurosyphilis—should always be based on a comprehensive evaluation that includes clinical findings, patient history, and additional diagnostic methods. High titers alone are insufficient for diagnosis, highlighting the necessity of thorough clinical assessment.

Regarding the continued use of the Indirect Hemagglutination Assay (IHA) in our setting, although many current guidelines recommend treponemal immunoassays or chemiluminescence immunoassays (CLIA) due to their higher sensitivity and automation, several practical factors influence the persistence of IHA testing. These include cost-effectiveness, ease of use in resource-limited settings, and local availability. Our institution continues to employ IHA alongside non-treponemal tests as part of the established diagnostic workflow, ensuring consistent longitudinal data comparability across the study period. While IHA has provided consistent and supportive results in our setting, ongoing evaluation and potential integration of more advanced treponemal assays are essential for improving diagnostic accuracy and patient management [1,5,29].

Taken together, our study supports the integration of stage-specific serological interpretation into national syphilis management protocols and highlights the need for improved surveillance systems to better reflect the true disease burden.

Interpreting the correlation between IHA titers and the clinical stage of syphilis is often challenging and, in many cases, of limited clinical significance. This is because IHA titration results can be significantly influenced by individual factors such as the patient’s immune status, the presence of HIV infection, or reinfection. Therefore, classifying or staging patients solely based on a specific IHA titer level—without knowledge of these underlying characteristics—can be misleading. These factors must be carefully considered in clinical assessments, and disease staging should not rely solely on serological titers.

The rising number of late latent syphilis cases suggests delays in diagnosis and highlights the need to improve early detection strategies. This upward trend, particularly noticeable after 2011 and peaking in 2024, is clearly illustrated in Figure 5.

The increase in primary and secondary cases indicates ongoing transmission and underscores the need for strengthened STI surveillance and prevention efforts. The recent occurrence of neurosyphilis and congenital syphilis further suggests critical gaps in adult treatment and prenatal screening services, calling for more comprehensive public health interventions.

Information regarding whether the cases in our study had previous syphilis infections was limited, which is thought to stem from social stigmas related to sexually transmitted infections in our country. Patients may be hesitant to disclose their transmission routes and prior treatment history or may seek care at different healthcare institutions, resulting in incomplete reporting of their infection histories. In our study, only the initial test results performed at our center were evaluated, and some cases were followed up over an extended period. However, the assessment of reinfections and detailed infection histories falls within the scope of infectious disease specialists and was not examined in detail in our study. Therefore, the lack of knowledge about previous syphilis infection history should be considered a significant limitation in the interpretation of our data.

The predominance of late latent syphilis cases in our study is related not only to its retrospective design but also to the fact that our center is a tertiary care institution. Since patients with diagnostic difficulties or those previously undiagnosed are frequently referred to our center at more advanced stages, the proportion of late-stage syphilis cases in our patient population was found to be high, and we believe some of these cases may have been misclassified. Similarly, Kohn et al. reported that a significant portion of early latent and unknown duration cases were misclassified [30]. Additionally, retrospective analyses conducted in Los Angeles and Alberta have shown that late latent cases are often confused with early latent or unknown duration cases [31,32]. These literature findings support the classification limitations in our study and emphasize the need for caution when interpreting the data. In particular, cases with low IHA titers and unknown prior history of syphilis or treatment may be subject to classification bias in diagnosing late latent syphilis. Physicians may have tended to classify these cases as late latent syphilis to avoid undertreatment; this could have led to misclassification of some cases and potential distortions in the interpretation of the results.

In our study, HIV-positive and HIV-negative individuals were evaluated in the same group, although it should be kept in mind that HIV status may affect pre-treatment RPR titers.

IHA or RPR titers alone appear to be insufficient for reliably determining the stage of syphilis. However, combined interpretation of these tests may offer supportive insights for clinical assessment, especially in settings where molecular diagnostic tools are limited. Nevertheless, given the retrospective design of the study, the potential for staging errors, the unknown history of previous syphilis infections, and changes in test kits over time, these findings should be interpreted with caution.

The stage-specific serological patterns observed in our dataset should be considered preliminary and not conclusive. They may serve as a basis for future, well-controlled studies. Prospective research is needed to determine whether antibody titer thresholds can reliably aid in disease staging, guide treatment decisions, or inform follow-up strategies.

### Limitations of the Study

This study has a retrospective design, and some patient records contain incomplete or limited data. The stage of the disease could not be definitively determined for all cases. As the study was conducted from a laboratory-based perspective, clinical data such as treatment history, immune status, HIV infection, and reinfection were not systematically evaluated. These aspects are being investigated in a separate study by the Department of Infectious Diseases.

Serological tests, particularly IHA titers, may vary in immunosuppressed, HIV-positive, or reinfected individuals. Therefore, serological findings are presented descriptively and without direct clinical interpretation.

While a conventional testing algorithm (RPR screening followed by IHA confirmation) is used in our institution, a reverse algorithm is employed at the affiliated blood center; data from that center were not included in this study.

Over the 20-year study period, the brands of test kits used have changed. Although such changes may have limited impact, they were managed through rigorous quality control and validation procedures. Nonetheless, potential effects of these variations should be considered when interpreting results.

Data on the frequency of syphilis testing during the study period were unavailable, making it impossible to assess how testing volume may have influenced the number of detected cases. Behavioral data such as sexual orientation and condom use were not reliably obtained and thus were not included, potentially limiting the interpretation of gender-related findings.

Cases of late latent syphilis diagnosed outside our institution may not have been fully captured, possibly affecting case ascertainment. Additionally, case numbers were reported in absolute terms and not normalized to outpatient visit volume or regional population, which may introduce surveillance bias.

Primary and secondary syphilis cases were less common compared to late latent cases, likely due to the retrospective nature of the study and the tertiary referral status of our institution. Patients often presented at later stages due to delayed or missed diagnoses. Incomplete clinical data also pose a risk of misclassification. Similar inconsistencies in the differentiation between early and late latent syphilis have been reported in the literature.

Finally, previous syphilis infection or reinfection history could not be fully determined, likely due to social concerns or incomplete reporting from patients who had sought care at different healthcare facilities.

## 5. Conclusions

The total number of syphilis cases increased from 2 in 2005 to 30 in 2016 and 71 in 2024, representing approximately a 15-fold increase between 2005 and 2016 and a 35-fold increase between 2005 and 2024. The rise among young males after 2018 is particularly striking. The high prevalence of HIV coinfection among males highlights the increased risk of concurrent transmission and the need for a multidisciplinary approach to clinical management. Our findings indicate a regional increase in syphilis cases and emphasize the necessity for ongoing surveillance and targeted public health interventions. Effective measures should be implemented to address transmission routes, develop strategies specific to risk groups, and update screening programs.

However, changes in case finding strategies, such as expanded prenatal screening programs and increased outreach to high-risk groups like men who have sex with men (MSM), may also have contributed to the observed rise in detected cases.

Serological test results, consistent with clinical guidelines, demonstrate that IHA and RPR tests are important diagnostic tools for determining syphilis stages.

Our study, as a single-center retrospective analysis, has limited generalizability. Future multicenter, prospective studies can more comprehensively assess the epidemiological trends of syphilis.

## Figures and Tables

**Table 1:** Sociodemographic and transmission characteristics of syphilis cases by gender. Column percentages were calculated based on the total number of cases within each gender group: men (*n* = 501), women (*n* = 170), and total (*n* = 671). Percentages are presented as number (%).

Group	Subcategory	Men (*n* = 501)	Women (*n* = 170)	Total (*n* = 671)
**Transmission Route**	**Heterosexual Contact**	376 (75)	100 (58.8)	**476 (70.9)**
	**Blood**	10 (2)	8 (4.7)	**18 (2.7)**
	**Substance Use**	14 (2.8)	1 (0.6)	**15 (2.2)**
	**Mother-to-Child**	8 (1.6)	10 (5.9)	**18 (2.7)**
	**Unknown**	93 (18.6)	51 (30)	**144 (21.5)**
**Place of Residence**	**Bursa**	457 (91.2)	153 (90)	**610 (91)**
	**Outside of Bursa**	44 (8.8)	17 (10)	**61 (9)**
**Marital Status**	**Married**	228 (45.5)	106 (62.3)	**334 (49.8)**
	**Single**	265 (52.9)	54 (31.8)	**319 (47.5)**
	**Under 18**	8 (1.6)	10 (5.9)	**18 (2.7)**
**Disease Stage**	**Primary**	25 (3.7%)	4 (0.6%)	**29 (4.3%)**
	**Secondary**	91 (13.6%)	20 (3.0%)	**110 (16.4%)**
	**Early Latent**	14 (2.1%)	2 (0.3%)	**16 (2.4%)**
	**Late Latent**	350 (52.2%)	134 (20.0%)	**485 (72.3%)**
	**Neurosyphilis**	13 (1.9%)	0 (0.0%)	**13 (1.9%)**
	**Congenital Syphilis**	8 (1.2%)	10 (1.5%)	**18 (2.7%)**

**Table 2:** Distribution of HIV, HBV, and HCV coinfections in syphilis cases by gender.

	Women (*n* = 170)	Man (*n* = 501)	χ^2^	df	*p*	Cramer’s V
**HIV**	6 (3.5)	213 (42.5)	87.747	1	<0.001	0.362
**HBV**	2 (1.2)	6 (1.2)	-	-	1.000 *	0.001
**HCV**	2 (1.2)	15 (3)	-	-	0.263 *	0.050
**HIV + HCV**	0 (0)	3 (0.6)	-	-	0.575 *	0.039
**HIV + HBV**	0 (0)	4 (0.8)	-	-	0.577 *	0.045
**HCV + HBV**	1 (0.6)	0 (0)	-	-	1.000 *	0.066
**HIV + HCV + HBV**	0 (0)	0 (0)	-	-	-	-

* Fisher’s exact test was used.

## Data Availability

All data and materials are available, and the corresponding author is Payaslıoğlu M.

## Abbreviations

The following abbreviations are used in this manuscript:

HA	Indirect Hemagglutination Assay
RPR	Rapid Plasma Reagin
HIV	Human Immunodeficiency Virus
MSM	Men Who Have Sex with Men
CDC	Centers for Disease Control and Prevention
CLIA	Chemiluminescence Immunoassay
HBV	Hepatitis B Virus
HCV	Hepatitis C Virus
